# Efficacy and safety of B-mode ultrasound-guided percutaneous transhepatic gallbladder drainage combined with laparoscopic cholecystectomy for acute cholecystitis in elderly and high-risk patients

**DOI:** 10.1186/s12876-015-0294-2

**Published:** 2015-07-09

**Authors:** Yi-Ren Hu, Jiang-Hua Pan, Xiao-Chun Tong, Ke-Qin Li, Sen-Rui Chen, Yi Huang

**Affiliations:** Department of General Surgery, Wenzhou People’s Hospital, The Third Clinical College of Wenzhou Medical University, No. 57 Canghou Street, Wenzhou, 325000 P.R. China

**Keywords:** Percutaneous transhepatic gallbladder drainage, Laparoscopic cholecystectomy, Acute cholecystitis, B-mode ultrasound, Efficacy, Safety

## Abstract

**Background:**

Standards in treatment of acute cholecystitis (AC) in the elderly and high-risk patients has not been established. Our study evaluated the efficacy and safety of B-mode ultrasound-guided percutaneous transhepatic gallbladder drainage (PTGD) in combination with laparoscopic cholecystectomy (LC) for acute cholecystitis (AC) in elderly and high-risk patients.

**Methods:**

Our study enrolled 35 elderly and high-risk AC patients, hospitalized between January 2010 and April 2014 at the Wenzhou People's Hospital. The patients underwent B-mode ultrasound-guided PTGD and LC (PTGD + LC group). As controls, a separate group of 35 elderly and high-risk AC patients who underwent LC alone (LC group) during the same period at the same hospital were randomly selected from a pool of 186 such cases. The volume of bleeding, surgery time, postoperative length of stay, conversion rate to laparotomy and complication rates (bile leakage, bleeding, incisional hernia, incision infection, pulmonary infarction and respiratory failure) were recorded for each patient in the two groups.

**Results:**

All patients in the PTGD + LC group successfully underwent PTGD. In the PTGD + LC group, abdominal pain in patients was relieved and leukocyte count, alkaline phosphatase level, total bilirubin and carbohydrate antigen 19-9 (CA19-9) decreased to normal range, and alanine aminotransferase and aspartate aminotransferase levels improved significantly within 72 h after treatment. All patients in the PTGD + LC group underwent LC within 6–10 weeks after PTGD. Our study revealed that PTGD + LC showed a significantly higher efficacy and safety compared to LC alone in AC treatment, as measured by the following parameters: duration of operation, postoperative length of hospital stay, volume of bleeding, conversion rate to laparotomy and complication rate (operation time of LC: 55.6 ± 23.3 min vs. 91.35 ± 25.1 min; hospitalized period after LC: 3.0 ± 1.3 d vs. 7.0 ± 1.7 d; intraoperative bleeding: 28.7 ± 15.2 ml vs. 60.38 ± 16.4 ml; conversion to laparotomy: 3 cases vs. 10 cases; complication: 3 cases vs. 8 cases; all *P* < 0.05 ).

**Conclusion:**

Our results suggest that B-mode ultrasound-guided PTGD in combination with LC is superior to LC alone for treatment of AC in elderly and high-risk patients, showing multiple advantages of minimal wounding, accelerated recovery, higher safety and efficacy, and fewer complications.

## Background

Acute cholecystitis (AC) is an inflammatory gallbladder disease caused by the obstruction and bacterial invasion of the cystic duct, and more than 90 % of AC cases are associated with cholelithiasis or gallstones [1]. The incidence of AC increases with age and shows dramatic gender differences, at 11.5 % in men and 22.4 % in women by age 60, and AC is the most common cause of acute abdominal surgeries in the elderly [2, 3]. Elderly AC patients have higher postoperative mortality and morbidity rates, increased complication rates, longer length of hospital stays and longer recovery times [4]. In most AC patients, laparoscopic cholecystectomy (LC) can rapidly relieve the signs and symptoms of inflammation within 48–72 h [5, 6]. Nevertheless, in elderly AC patients with other life-threatening comorbidities, considered as the high-risk AC group, LC can lead to high morbidity of up to 41 % and mortality of up to 4.5 % during the acute phase [7, 8]. In case of severe or complex cholecystitis in elderly AC patients, LC treatment is associated with higher rates of conversion to open cholecystectomy, with increased postoperative complications and longer lengths of hospital stay [9, 10]. For example, in elderly and high-risk AC patients, the perioperative mortality rate is high at 19 % for emergency cholecystectomy. Further, in several instances in elderly and high-risk patients, it is difficult to complete LC procedure because of severe pericholecystic inflammation.

Percutaneous transhepatic gallbladder drainage (PTGD) is a minimally invasive image-guided procedure designed to decompress the acutely inflamed gallbladder by draining the bile or pus from the bile duct using percutaneous liver gallbladder puncture catheter [11, 12]. PTGD is a palliative therapy recommended as an first-lone therapeutic choice in the elderly and high risk AC patients, and is performed as a simple operation, with low requirement for sophisticated equipment, lesser trauma and pain to the patients, and high efficiency and quick recovery [13–16]. The most frequent imaging guide tool for PTGD is ultrasound, which provides images in real-time without the risks of radiation, and is highly economical [17]. A few previous studies observed that when LC is preceded by PTGD, there were minimal rates of conversion and low perioperative morbidity and mortality in high-risk AC patients [18–20]. Based on the previous studies, we hypothesized that elderly and high-risk AC patients may derive significant clinical benefit from PTGD under the guidance of B-mode ultrasound and followed by LC [21–23]. In this study, we test our hypothesis by investigating the clinical value of sequentially combining PTGD under the guidance of B-ultrasound with LC as a minimally invasive treatment procedure in elderly and high-risk AC patients.

## Methods

### Ethics statement

The study was approved by the Institutional Review Board of the First People’s Hospital of Wenzhou, China. The informed written consent was collected from each eligible patient and the whole study was performed based on the guidelines and principles of the Declaration of Helsinki [24].

### Study subjects

This study enrolled 35 elderly (all > 65 years old) and high-risk AC patients who underwent B-mode ultrasound-guided PTGD combined with LC (PTGD + LC group) at Ethe First People’s Hospital of Wenzhou between January 2010 and April 2014. A separate group of 35 elderly and high-risk AC patients were randomly selected from a pool of 186 cases who underwent only LC (LC group) during the same period at the same hospital. The PTGD + LC group contained 23 males and 12 females, with a mean age of 69.2 ± 11.4 years. The LC group contained 20 males and 15 females, with a mean age of 71.5 ± 11.5 years. The diagnostic criteria and evaluation of severity were based on the revised Tokyo guidelines 2012, namely, local signs of inflammation (Murphy’s sign and right upper quadrant mass/pain/tenderness), systemic symptoms of inflammation (fever, elevated white blood cell count and elevated C-reactive protein), and findings from computed tomography (CT) scans and ultrasonograms [25, 26]. By admission time, the onset period in patients ranged from 6 h to 72 h, with an average of 30 h. All the patients had upper abdominal pain or right upper abdominal pain. Among the total 70 patients in this study, 46 cases had fever, 54 cases had shoulder and back radiating pain and all patients exhibited right upper abdominal tenderness by physical examination. A total of 51 cases showed positive Murphy’s sign and 40 cases had palpable and swollen gallbladder with tenderness. Leukocytes count in all patients were elevated and were in the 2.2 ± 0.5 grade, based on the American Society of Anesthesiologists (ASA) grade. Gallbladder swelling and wall thickening were found using liver and gallbladder B-ultrasound and 26 cases had pericholecystic fluid and 63 cases had cholecystolithiasis. All subjects in the LC and PTGD + LC groups met the following study inclusion and exclusion criteria. Inclusion criteria: (1) elderly AC patients (>65 years old) with cardiac, brain and lung comorbidities, and unable to tolerate surgery; (2) patients with AC and empyema of gall-bladder, failing to response well to non-surgical therapies; (3) severe AC patients with disease for more than 48–72 h, diameter of gallbladder enlargement 8 cm, and gallbladder wall of thickening 4 mm. Exclusion criteria: (1) patients with dysfunction in blood coagulation and severe bleeding tendency; (2) patients with massive ascites; (3) patients with gallbladder in free state; (4) patients’ gallbladder with clear or inappropriate puncture route; (5) patients with general peritonitis and suspected perforation of gallbladder.

### PTGD treatment

All patients received routine blood, urine and feces examination and biochemical tests. Patients were given routine fluid replacement to balance fluids, electrolytes, and to maintain acid–base equilibrium and nutrition. Treatments for infections were given if routine blood examination supported such intervention and the patients with hemorrhagic shock were managed by quickly controlling the source of bleeding and resuscitation [27]. PTGD + LC in elderly high-risk AC patients was accomplished by PTGD guided by B-Ultrasound. Patients were primarily scanned by B-Ultrasound in right lateral decubitus or supine position and the puncture plan was simulated based on the image, with corresponding marks on the patient. After local disinfection and draping, 5 % lidocaine (5 ml) local anesthesia was administered and an incision of 2 ~ 3 mm was made. Using B-Ultrasound guidance, an 18-gauge puncture needle (Angiotech Company, Denmark) was advanced transhepatically into the gallbladder. After placing a guide wire and dilating the track, a 6Fr ~ 10Fr pigtail catheter was positioned with its tip in the gallbladder, followed by pull out the guide wire and placing the drainage bag. After PTGD surgery, the drainage tube was washed with metronidazole injection twice a day and patients were observed for pneumothorax, bile peritonitis and other complications. In AC patients with cardiovascular and cerebrovascular diseases [28], the systolic blood pressure (SBP) in hypertensive patients was controlled under 140mmHG. In AC patients with diabetes mellitus [29], fasting glucose was controlled within 4.5 ~ 8.0 mmol/L. In patients with cardiac insufficiency [30], the heart function grade was kept under grade 2. In patients with asthma [31], chronic bronchitis [32] and other respiratory diseases, the infection status was monitored and appropriately controlled, and anti-inflammatory and phlegm elimination was employed, with maximal ventilatory volume over 60 ~ 70 %.

### PTGD postoperative index

The patient’s condition after PTGD was evaluated: (1) symptoms: abdominal pain grade, body temperature; (2) body signs: abdominal tenderness; (3) laboratory examination: leukocyte counts, alkaline phosphatase (ALP), alanine transaminase (ALT), aspartate aminotransferase (AST), total bilirubin (TB) and carbohydrate antigen 19-9 (CA19-9); reexamination of liver and gallbladder by the B-ultrasound after surgery for two weeks to detect the gallbladder wall thickness and pericholecystic fluid situation. Visual analogue scale (VAS) was adopted for pain grading. After achieving symptomatic relief, patients in PTGD + LC group were given LC within 6 ~ 10 weeks (average of 8 weeks) after PTGD.

### LC treatment

A cannula (10 mm) was placed on the upper edge of umbilicus of the patients for inspection, in which 30° laparoscope was set to observe the gallbladder and its surrounding area. A 10 mm cannula was also placed below xiphoid bone under direct vision and two cannula (5 mm) were placed in right upper quadrant. Subsequently, the cystohepatic triangle of patients, with head up in left lateral position, was separated. The gallbladder was stripped off the liver with electrocautery. Patients were intramuscularly injected with Hemocoagulase 1U (Zhaoke Pharmaceutical (Hefei) Co., Ltd., China) for 2 days, each day for 8 h. Hemocoagulase is a proteolytic enzyme used as a plasma clotting agent for fibrinogen and for detection of fibrinogen degradation products. It is a hemostatic drug used to treat primary and secondary bleeding in postoperative period. Patients were also treated immediately if their blood glucose and blood pressure changed significantly during transfusion.

### LC postoperative evaluation index

All patients enrolled in this study were informed of the benefits and risks of both interventions. All interventions were provided by an experienced medical team with more than 10 years of clinical and surgical experience. The team included surgeon, an emergency physician and a gastroenterologist. After LC surgery, data was collected from the patients in PTGD + LC group and LC group and the following parameters were compared: the duration of the surgery, the amount of intraoperative bleeding, conversion rate to laparotomy, postoperative complication rate, mortality, length of stay. VAS was employed to grade pain.

### Statistical analysis

Statistical analysis was performed using SPSS 17.0 software (SPSS Inc., Chicago, IL, USA). Continuous data were expressed as mean ± standard deviation (SD) and further analyzed by *t* test. Categorical data in the study was tested using χ^2^ test. *P* < 0.05 indicated statistically significant results.

## Results

### Baseline characteristics

As shown in Table 1, the differences in age, gender, ASA grade, abdominal pain grade, body temperature (°C), positive Murphy’s sign, leukocytes counts and B-ultrasound gallbladder wall thickness showed no statistically significant differences between the PTGD + LC group and LC group (all *P* > 0.05). All 35 patients in the PTGD + LC group had internal diseases (100 %) while 32 patients in the LC group had internal diseases (91.4 %), and this difference in patients with internal diseases between the two groups was not statistically significant (*P* = 0.077).

**Table 1:** The baseline characteristics of the patients in PTGD + LC group and LC group

	PTGD + LC group (*n* = 35)	LC group (*n* = 35)	*P*
Age	69.2 ± 11.4	71.5 ± 11.5	0.404
Gender (M/F)	23/12	20/15	0.461
Comorbidities (%)	35 (100.0 %)	32 (91.4 %)	0.077
Hypertension	19	15	
Diabetes	14	14	
Arhythmia	2	4	
CHD	7	4	
COPD	9	3	
CI	0	1	
CRI	0	1	
ASA grade (average)	2.2 ± 0.6	2.2 ± 0.5	1.000
Abdominal pain grade	5.1 ± 1.5	4.9 ± 1.4	0.566
Body temperature (°C)	38.1 ± 0.8	37.8 ± 0.6	0.081
Positive Murphy’s sign	25 (71.4 %)	26 (74.3 %)	0.072
Leukocytes counts (per/ml)	12.3 ± 3.7	12.7 ± 3.5	0.644
Gallbladder wall thickness (cm)	0.68 ± 0.34	0.71 ± 0.28	0.688

*M*: male; *F*: female; *PTGD*: percutaneous transhepatic gallbladder drainage; *LC*: laparoscopic cholecystectomy; *CHD*: coronary heart disease; *COPD*: chronic obstructive pulmonary disease; *CI*: cerebral infarction; *CRI*: chronic renal insufficiency; *ASA grade*: American Society of Anesthesiologists grade

### Postoperative situation

All 35 patients underwent PTGD successfully with an average operation time of 50.4 (40 ~ 75) min. There were 24 cases (68.6 %) of positive bile bacteria cultures, including *E.coli* and other bateria (Klebsiella pneumoniae, Enterococcus faecium, Pseudomonas aeruginosa), and antibacterial drugs were used for treatment for an average time of 4.3 ± 2.6d. As shown in Table 2, within 72 h after PTGD surgery, patients’ body temperature was restored to normalcy, and leukocyte counts, ALP , TB and CA19-9 levels were also within the normal range, with significant improvement compared to preoperative levels (all *P* < 0.05). ALT and AST levels improved significantly when compared with the preoperative levels (*P* < 0.05). PTGD postoperative abdominal pain grading was lower than the preoperative grading (2.3 ± 0.9 vs. 5.1 ± 1.5, *P* < 0.001). The patients were reexamined 4 weeks after PTGD surgery and thinner gallbladder wall (0.34 ± 0.20) was noted by B-ultrasound of liver and gallbladder, and ocal inflammation of gallbladder was significantly reduced. There were 3 cases of postoperative complications after PTGD surgery. Of the 3 cases, 2 cases had their ducts dislodged and one case involved bleeding. All PTGD + LC group patients underwent LC surgery within 6–10 weeks (average of 8 weeks) after PTGD surgery.

**Table 2:** Clinical manifestations and alterations of laboratory examination within 72 h after percutaneous transhepatic gallbladder drainage surgery

	Before PTGD	After PTGD	*P*
Abdominal pain grade	5.1 ± 1.5	2.3 ± 0.9	<0.001
Body temperature (°C)	38.1 ± 0.8	36.5 ± 1.5	<0.001
Leukocytes counts (×10^9^/L)	12.3 ± 3.7	6.7 ± 3.5	<0.001
ALP (U/L)	144.3 ± 32.6	104.5 ± 16.7	<0.001
ALT (U/L)	74.9 ± 35.2	48.9 ± 24.0	<0.001
AST (U/L)	81.6 ± 41.2	39.6 ± 18.3	<0.001
TB (μmol/L)	42.3 ± 15.2	22.5 ± 11.4	<0.001
CA19-9 (U/mL)	620.5 ± 205.6	55.2 ± 20.1	<0.001

*PTGD*: percutaneous transhepatic gallbladder drainage; *ALP*: alkaline phosphatase; *ALT*: alanine transaminase; *AST*: aspartate aminotransferase; *TB*: total bilirubin; *CA19-9*: carbohydrate antigen 19-9

### Operative indexes

As shown in Table 3, the duration of LC surgery in PTGD + LC group was 55.6 ± 23.3 min, while it was 91.4 ± 25.1 min in the LC group. The length of hospital stay of the PTGD + LC group after LC surgery was 3.0 ± 1.3 d, while it was 7.0 ± 1.7 d in the LC group. The differences of operative duration and length of hospital stay was statistically significant between the two groups (*P* < 0.05 for both). Intraoperative bleeding of the patients in PTGD + LC group (28.7 ± 15.2 ml) was significantly lower than observed in patients of the LC group (60.4 ± 16.4 ml), and the differences were statistically significant (*P* < 0.001). Three cases in PTGD + LC group (8.6 %) and 10 cases in LC group converted to laparotomy because of severe adhesion of the gallbladder triangle or difficult exposure of the gallbladder. The conversion rate to laparotomy rate of the PTGD + LC group was significantly lower than the LC group (*P* = 0.031).

**Table 3:** Comparison of observation indexes of laparoscopic cholecystectomy in patients between PTGD + LC group and LC

	PTGD + LC group (*n* = 35)	LC group (*n* = 35)	*P*
Operative duration (min)	55.6 ± 23.3	91.4 ± 25.1	<0.01
The amount of intraoperative bleeding (ml)	28.7 ± 15.2	60.4 ± 16.4	<0.01
Conversion rate to laparotomy (%)	3 (8.6 %)	10 (28.6 %)	0.031
Hospital length of stay (d)	3.0 ± 1.3	7.0 ± 1.7	<0.01

*PTGD*: percutaneous transhepatic gallbladder drainage, *LC*: laparoscopic cholecystectomy

### Postoperative complications

There were no deaths reported in the two groups. There was 1 case of bile leakage and 1 case of wound infection in the PTGD + LC group, showing a 5.7 % rate of postoperative complications (2/35). There were 2 cases of bile leakage, 2 cases of wound infection and 1 case each of bleeding, hernia, pulmonary infarction and respiratory failure in the LC group, showing a 22.9 % rate of postoperative complications (8/35). The postoperative complications rate of PTGD + LC group was significantly lower than the LC group (χ^2^ = 4.20, *P* = 0.040).

## Discussion

LC is a highly effective surgical procedure in AC patients. However, in elderly and high-risk patients with other severe co-existing illnesses, higher mortality rates and higher conversion rate to an open procedure is observed during the acute phase [33, 34]. Therefore, elderly and high-risk AC patients are in urgent need of improved procedures and techniques to safely manage the disease, which is important considering the rapidly growing elderly populations worldwide [35]. Towards this goal, this study investigated the efficacy and safety of B-mode ultrasound-guided PTGD in combination with LC in elderly and high-risk AC patients.

The main finding in our study is that B-ultrasound guided PTGD, combined with LC, is superior to LC alone for effective treatment of elderly and high-risk AC patients. AC is caused by an obstruction of cystic duct and the drainage of gallbladder bile is an important component in treatment of AC to relieve pain and reduce inflammation [36]. The use of PTGD prior to LC has the following benefits: first, PTGD is a simple, local and a minimally invasive procedure used in patients with severe systemic diseased, which makes them unsuitable for LC [37]. Second, PTGD is excellent for decompression of swollen gallbladder and prevents gallbladder necrosis and perforation, achieving improved local circulation and control of infection. The congestive bile can be quickly be released from the body by puncturing and the clinical symptoms of AC in patients are promptly relieved by this procedure [38]. In our study, all 35 patients underwent PTGD successfully. After the infected bile was drained, the inflamed gallbladder was decompressed and the body temperature in all 35 patients decreased to normal levels within 72 h. All patients reported dramatically reduced abdominal pain and their leukocyte counts were within normal range, with the levels of ALP, TB and CA19-9 also decreased to normal range. Third, patients can receive antibiotic therapy to relieve the symptoms of infection such as fever and abdominal pain, after PTGD [39]. In our study, we encountered 24 cases of positive bile bacteria cultures, including *E.coli* and other bateria such as Klebsiella pneumoniae, Enterococcus faecium, Pseudomonas aeruginosa, and subsequently, antibacterial drugs were used in these patients. Fourth, PTGD can be employed for cholangiography, which is crucial for revealing the biliary tract anatomy and for selection of therapy options. Preoperative cholangiography, using PTGD tube, can provide a clear information on the bile duct, cystic duct and gallstone, and additionally intraoperative cholangiography can greatly avoid injury [40]. Finally, PTGD allows the choice of subsequent elective cholecystectomy, with minimal rates of conversion, and is considered as a bridge in the elderly and high-risk patient population, prior to LC [19]. In our study, all 35 patients had LC surgery within 6–10 weeks after PTGD surgery and these patients had lower conversion rate to laparotomy and reduced postoperative complications rates, compared with the patients who underwent LC alone. B-mode is an accurate and non-invasive imaging modality and is highly specific for detecting biliary obstruction and gallstones. The segmental and subsegmental branches of both the hepatic artery and the portal vein can be identified and discriminated from bile ducts in B-mode [41]. Xu EJ *et al.* also demonstrated that PTGD is more successful and efficacious when guided by ultrasound imaging [17]. Both Kim *et al.* and Shibasaki *et al.* demonstrated that PTGD is a less invasive image-guided alternative designed to decompress the acutely inflamed gallbladder in patients who are unresponsive to medical therapy or are at high risk for cholecystectomy [18, 42].

## Conclusion

In conclusion, our study provides strong evidence that B-mode ultrasound-guided PTGD, combined with LC, is superior to LC alone for treatment of AC in elderly and high-risk patients. The advantages include minimal wound, accelerated recovery, higher safety and efficacy, and fewer complications. However, larger prospective randomized trials are needed to confirm these findings.

## Abbreviations

PTGD: Percutaneous transhepatic gallbladder drainage
LC: Laparoscopic cholecystectomy
ASA: American Society of Anesthesiologists
VAS: Visual analogue scale

## References

[1] Rogers PN (2014). Long-term outcome of patients with acute cholecystitis receiving antibiotic treatment: a retrospective cohort study. World J Surg.

[2] Rodriguez-Sanjuan JC, Arruabarrena A, Sanchez-Moreno L, Gonzalez-Sanchez F, Herrera LA, Gomez-Fleitas M. Acute cholecystitis in high surgical risk patients: percutaneous cholecystostomy or emergency cholecystectomy? Am J Surg. 2012;204(1):54–9. 10.1016/j.amjsurg.2011.05.013 22000114

[3] Cull JD, Velasco JM, Czubak A, Rice D, Brown EC. Management of acute cholecystitis: prevalence of percutaneous cholecystostomy and delayed cholecystectomy in the elderly. J Gastrointest Surg. 2014;18(2):328–33. 10.1007/s11605-013-2341-z 24197550

[4] Bueno Lledo J, Vaque Urbaneja J, Herrero Bernabeu C, Castillo Garcia E, Carbonell Tatay F, Baquero Valdelomar R, et al. Acute cholecystitis and laparoscopic cholecystectomy in the elderly. Cir Esp. 2007;81(4):213–7. 10.1016/s0009-739x(07)71302-7 17403358

[5] Li M, Li N, Ji W, Quan Z, Wan X, Wu X, et al. Percutaneous cholecystostomy is a definitive treatment for acute cholecystitis in elderly high-risk patients. Am Surg. 2013;79(5):524–7. 10.1177/000313481307900529 23635589

[6] Kirshtein B, Bayme M, Bolotin A, Mizrahi S, Lantsberg L. Laparoscopic cholecystectomy for acute cholecystitis in the elderly: is it safe? Surg Laparosc Endosc Percutan Tech. 2008;18(4):334–9. 10.1097/SLE.0b013e318171525d 18716529

[7] Chok KS, Chu FS, Cheung TT, Lam VW, Yuen WK, Ng KK, et al. Results of percutaneous transhepatic cholecystostomy for high surgical risk patients with acute cholecystitis. ANZ J Surg. 2010;80(4):280–3. 10.1111/j.1445-2197.2009.05105.x 20575957

[8] Kortram K, van Ramshorst B, Bollen TL, Besselink MG, Gouma DJ, Karsten T, et al. Acute cholecystitis in high risk surgical patients: percutaneous cholecystostomy versus laparoscopic cholecystectomy (CHOCOLATE trial): study protocol for a randomized controlled trial. Trials. 2012;13:7. 10.1186/1745-6215-13-7 PMC3285056 22236534

[9] To KB, Cherry-Bukowiec JR, Englesbe MJ, Terjimanian MN, Shijie C, Campbell Jr DA, et al. Emergent versus elective cholecystectomy: conversion rates and outcomes. Surg Infect (Larchmt). 2013;14(6):512–9. 10.1089/sur.2012.160 24274058

[10] Dolan JP, Diggs BS, Sheppard BC, Hunter JG. The national mortality burden and significant factors associated with open and laparoscopic cholecystectomy: 1997–2006. J Gastrointest Surg. 2009;13(12):2292–301. 10.1007/s11605-009-0988-2 19727976

[11] Oku T, Horii T, Masaka T, Miseki T, Sakai T, Kamiga T. Clinical comparison of endoscopic naso-gallbladder drainage versus percutaneous transhepatic gallbladder drainage for acute cholecystitis. Nihon Shokakibyo Gakkai Zasshi. 2013;110(6):989–97. 23739731

[12] Yu W, Li W, Wang Z, Ye X, Li N, Li J. Early percutaneous transhepatic gallbladder drainage compared with endoscopic retrograde cholangiopancreatography and papillotomy treatment for severe gallstone associated acute pancreatitis. Postgrad Med J. 2007;83(977):187–91. 10.1136/pgmj.2006.047746 PMC2599991 17344574

[13] Tian H, Xia M, Zhang S, Li J, Liu J. Acute calculous cholecystitis associated with hepatic artery pseudoaneurysm after percutaneous transhepatic gallbladder drainage in a diabetic patient. Chin Med J (Engl). 2014;127(17):3192–4. 25189970

[14] Zhao XQ, Dong JH, Jiang K, Huang XQ, Zhang WZ. Comparison of percutaneous transhepatic biliary drainage and endoscopic biliary drainage in the management of malignant biliary tract obstruction: A meta-analysis. Dig Endosc. 2014;27(1):137–45. 10.1111/den.12320 25040581

[15] Fujita T, Tanabe M, Takahashi S, Iida E, Matsunaga N. Percutaneous transhepatic hybrid biliary endoprostheses using both plastic and metallic stents for palliative treatment of malignant common bile duct obstruction. Eur J Cancer Care (Engl). 2013;22(6):782–8. 10.1111/ecc.12088 23834370

[16] Komatsu S, Tsukamoto T, Iwasaki T, Toyokawa A, Hasegawa Y, Tsuchida S, et al. Role of percutaneous transhepatic gallbladder aspiration in the early management of acute cholecystitis. J Dig Dis. 2014;15(12):669–75. 10.1111/1751-2980.12198 25233857

[17] Xu EJ, Zheng RQ, Su ZZ, Li K, Ren J, Guo HY. Intra-biliary contrast-enhanced ultrasound for evaluating biliary obstruction during percutaneous transhepatic biliary drainage: a preliminary study. Eur J Radiol. 2012;81(12):3846–50. 10.1016/j.ejrad.2012.06.025 22835875

[18] Shibasaki S, Takahashi N, Toi H, Tsuda I, Nakamura T, Hase T, et al. Percutaneous transhepatic gallbladder drainage followed by elective laparoscopic cholecystectomy in patients with moderate acute cholecystitis under antithrombotic therapy. J Hepatobiliary Pancreat Sci. 2014;21(5):335–42. 10.1002/jhbp.28 24027011

[19] Kim IG, Kim JS, Jeon JY, Jung JP, Chon SE, Kim HJ, et al. Percutaneous transhepatic gallbladder drainage changes emergency laparoscopic cholecystectomy to an elective operation in patients with acute cholecystitis. J Laparoendosc Adv Surg Tech A. 2011;21(10):941–6. 10.1089/lap.2011.0217 22129145

[20] Kim JH, Kim JW, Jeong IH, Choi TY, Yoo BM, Kim JH, et al. Surgical outcomes of laparoscopic cholecystectomy for severe acute cholecystitis. J Gastrointest Surg. 2008;12(5):829–35. 10.1007/s11605-008-0504-0 18327625

[21] Tsumura H, Ichikawa T, Hiyama E, Kagawa T, Nishihara M, Murakami Y, et al. An evaluation of laparoscopic cholecystectomy after selective percutaneous transhepatic gallbladder drainage for acute cholecystitis. Gastrointest Endosc. 2004;59(7):839–44. 10.1016/s0016-5107(04)00456-0 15173798

[22] Zhang GY, Li WT, Peng WJ, Li GD, He XH, Xu LC. Clinical outcomes and prediction of survival following percutaneous biliary drainage for malignant obstructive jaundice. Oncol Lett. 2014;7(4):1185–90. 10.3892/ol.2014.1860 PMC3961454 24944690

[23] Castro PM, Akerman D, Munhoz CB, Sacramento I, Mazzurana M, Alvarez GA. Laparoscopic cholecystectomy versus minilaparotomy in cholelithiasis: systematic review and meta-analysis. Arq Bras Cir Dig. 2014;27(2):148–53. 10.1590/S0102-67202014000200013 PMC4678672 25004295

[24] Holt GR. Declaration of Helsinki-the world's document of conscience and responsibility. South Med J. 2014;107(7):407. 10.14423/SMJ.0000000000000131 25010578

[25] Yokoe M, Takada T, Strasberg SM, Solomkin JS, Mayumi T, Gomi H, et al. New diagnostic criteria and severity assessment of acute cholecystitis in revised Tokyo Guidelines. J Hepatobiliary Pancreat Sci. 2012;19(5):578–85. 10.1007/s00534-012-0548-0 PMC3429769 22872303

[26] Han IW, Jang JY, Kang MJ, Lee KB, Lee SE, Kim SW. Early versus delayed laparoscopic cholecystectomy after percutaneous transhepatic gallbladder drainage. J Hepatobiliary Pancreat Sci. 2012;19(2):187–93. 10.1007/s00534-011-0458-6 21938408

[27] Sharma P, Benford B, Karaian JE, Keneally R. Effects of volume and composition of the resuscitative fluids in the treatment of hemorrhagic shock. J Emerg Trauma Shock. 2012;5(4):309–15. 10.4103/0974-2700.102372 PMC3519043 23248499

[28] Fuster V, Bansilal S (2010). Promoting cardiovascular and cerebrovascular health. Stroke.

[29] American Diabetes A. Diagnosis and classification of diabetes mellitus. Diabetes Care. 2014;37(1):S81–90. 10.2337/dc14-S081 24357215

[30] Wetsch WA, Lahm T, Hinkelbein J, Happel CM, Padosch SA. Cardiac insufficiency: acute right heart failure. Anasthesiol Intensivmed Notfallmed Schmerzther. 2011;46(11–12):718–25. 10.1055/s-0031-1297178 22147609

[31] Brehm JM (2015). Toward improved diagnosis of early asthma. Am J Respir Crit Care Med.

[32] Ferre A, Fuhrman C, Zureik M, Chouaid C, Vergnenegre A, Huchon G, et al. Chronic bronchitis in the general population: influence of age, gender and socio-economic conditions. Respir Med. 2012;106(3):467–71. 10.1016/j.rmed.2011.12.002 22197577

[33] Gurusamy K, Samraj K, Gluud C, Wilson E, Davidson BR. Meta-analysis of randomized controlled trials on the safety and effectiveness of early versus delayed laparoscopic cholecystectomy for acute cholecystitis. Br J Surg. 2010;97(2):141–50. 10.1002/bjs.6870 20035546

[34] Johner A, Raymakers A, Wiseman SM (2013). Cost utility of early versus delayed laparoscopic cholecystectomy for acute cholecystitis. Surg Endosc.

[35] Gurusamy KS, Rossi M, Davidson BR (2013). Percutaneous cholecystostomy for high-risk surgical patients with acute calculous cholecystitis. Cochrane Database Syst Rev.

[36] Itoi T, Coelho-Prabhu N, Baron TH (2010). Endoscopic gallbladder drainage for management of acute cholecystitis. Gastrointest Endosc.

[37] Huang CC, Lo HC, Tzeng YM, Huang HH, Chen JD, Kao WF, et al. Percutaneous transhepatic gall bladder drainage: a better initial therapeutic choice for patients with gall bladder perforation in the emergency department. Emerg Med J. 2007;24(12):836–40. 10.1136/emj.2007.052175 PMC2658354 18029515

[38] Ihama Y, Fukazawa M, Ninomiya K, Nagai T, Fuke C, Miyazaki T. Peritoneal bleeding due to percutaneous transhepatic gallbladder drainage: An autopsy report. World J Hepatol. 2012;4(10):288–90. 10.4254/wjh.v4.i10.288 PMC3537766 23301117

[39] Jang JW, Lee SS, Song TJ, Hyun YS, Park do H, Seo DW, et al. Endoscopic ultrasound-guided transmural and percutaneous transhepatic gallbladder drainage are comparable for acute cholecystitis. Gastroenterology. 2012;142(4):805–11. 10.1053/j.gastro.2011.12.051 22245666

[40] Fidelman N, Kerlan Jr RK, Laberge JM, Gordon RL. Accuracy of percutaneous transhepatic cholangiography in predicting the location and nature of major bile duct injuries. J Vasc Interv Radiol. 2011;22(6):884–92. 10.1016/j.jvir.2011.02.007 21514840

[41] Popescu A, Sporea I (2010). Ultrasound examination of normal gall bladder and biliary system. Med Ultrason.

[42] Kim HO, Ho Son B, Yoo CH, Ho Shin J. Impact of delayed laparoscopic cholecystectomy after percutaneous transhepatic gallbladder drainage for patients with complicated acute cholecystitis. Surg Laparosc Endosc Percutan Tech. 2009;19(1):20–4. 10.1097/SLE.0b013e318188e2fe 19238061

